# Dietary supplementation with *Jasminum sambac* leaf powder: Effects on growth, hematology, and immune parameters in broiler chickens

**DOI:** 10.1016/j.psj.2024.104645

**Published:** 2024-12-05

**Authors:** Iram Maqsood, Samreen Sareer, Shafique Ur Rehman, Ayesha Hidayat, Madeeha Shah, Saira Awaz, Rong Ke

**Affiliations:** a College of Wildlife and protected Areas, Northeast Forestry university Harbin, 150040, China; b Department of Zoology, Shaheed Benazir Bhutto Women University Peshawar, 25000 Pakistan; c Department of Botany, Government College University, Faisalabad, Pakistan

**Keywords:** *Jasminum sambac*, Broiler chickens, Hematological profiles, Immunological, Growth performance

## Abstract

Food production needs to expand by 70% to keep up with the expected considerable increase in the world population by 2050. As a major source of high-quality protein, poultry products are essential to the world's food supply. Nonetheless, problems with industrial mechanization, pollution, animal welfare, and antibiotic resistance affect the poultry industry. The objective of this study was to assess the impact of adding *Jasminum sambac* leaf powder (JSLP) to the feed of broiler chickens on their growth performance, coliform bacterial levels, and immunological function. During a 42-day period, 100 day-old Ross broiler chicks were randomly allocated to one of four treatment groups, each consisting of 25 replicates: T0 (control) with no additives, T1 supplemented with 3% JSLP, T2 with 6% JSLP, and T3 with 9% JSLP. One-way ANOVA was used to determine statistical inference among groups. Feeding JSLP resulted in a significant increase in body weight (BW), body weight gain (BWG), and feed intake (P<0.05), while faecal coliforms were significantly reduced (P<0.01). Further, haemoglobin concentration and red blood cell counts (RBC) on days 14, 28, and 42, were significantly increased(P<0.01). Additionally, significant differences in differential leucocyte counts (DLC) were observed between the JSLP-treated groups (P<0.001). Overall, JSLP supplementation positively influenced broiler chicken growth indices and haematological profiles, though its impact on immunological parameters warrants further investigation.

## Introduction

The poultry business is a significant and rapidly expanding sector in the food industry. It is anticipated to raise its output by 121% by 2050 compared to its levels in 2005 (Alexandratos and Bruinsma, 2012). The impressive expansion is a result of both the rise in worldwide meat consumption and notable progress in industrialization and economic productivity on a global scale. From 1961 to 2019, the production of broiler chickens had a significant and rapid increase, growing from 9 million tons to 132 million tons (Ayalew et al., 2022). Poultry meat has emerged as a primary provider of top-notch protein, vitamins, and minerals in developed and developing countries, underscoring its crucial contribution to worldwide food security (Chowdhury and Morey, 2019).

Previously, antibiotics have been widely used in the production of broiler meat to boost growth performance, increase feed conversion efficiency, and helps in reducing the spread of infectious illnesses (Lillehoj et al., 2018). The antibiotics as a growth booster in the broiler diets has caused significant public health concerns, including the emergence of antibiotic-resistant bacteria. Legal actions have been taken to combat this issue, such as the European Union's ban on utilizing antibiotics as growth enhancers in 2005 and China's subsequent ban in 2020 (Masud et al., 2020). The poultry industry is under increasing pressure to adopt sustainable farming methods that eliminate the use of antibiotics because, consumers become more aware of antibiotic residues and antimicrobial resistance. So there is a need to adopt new transition without harming profitability and animal health (Kelly and Alworth, 2013).

Production of antibiotic-free chicken faces considerable difficulties, especially in underdeveloped countries where there is commonly a lack of biosafety measures and conventional agricultural techniques (Peric et al., 2009). Inappropriate use of antibiotics has led to the rapid proliferation of microorganisms that exhibit resistance to antibiotics. This strategy not only harms the well-being of animals but also causing risks to human health and the environment (Berman et al., 2023). There is an increasing number of researches focused on discovering natural alternatives to antibiotics that can enhance animal health, enhance growth performance, and decrease reliance on chemical feed additives (Ayalew et al., 2022) Therefore, it is very crucial to explore the phytogenic feed additives, such as essential oils, herbal extracts, and other plant-derived chemicals that offer antibacterial, antioxidant, and growth-promoting properties (Eevuri and Putturu, 2013). Natural compounds serve as a safer and more environmentally friendly substitute for synthetic antibiotics, which could enhance the nutritional value of poultry products and complement customer preferences for safer and more natural food choices (Lillehoj et al., 2018).

*Jasminum sambac*, commonly referred to as jasmine, is an herbal substitute that has medicinal properties because of its several pharmacological attributes. Previously *J. sambac* has been prominent for its antibacterial, anti-inflammatory, and antioxidant qualities and adopted as a traditional medicine. *Jasminum sambac* is packed with variety of bioactive substances that might affect how poultry health is manage (AlRashdi et al., 2012). The exploratory study shows that the plant contains phytoconstituents like linalool and benzyl acetate essential oils, as well as alkaloids, carbohydrates, terpenoids, phenols, flavonoids, tannins, saponins, proteins, and phytosterols. Furthermore, the primary volatile substances found in *Jasminum* include linalool, benzyl alcohol, Z-3-hexenyl benzoate, indole, and methyl anthranilate (Reshma et al., 2021). Ongoing researches have unlocked its ability to enhance the immune system, contribute in good digestion, and reduction in the intestinal pathogenic bacteria. Therefore, it has the potential to be used as a natural solution for the problems faced in antibiotic-free chicken production (Tsai et al., 2010).

Although there are many potential advantages of *J. sambac*, but there is a dearth of research on its application in the poultry industry. In the past, traditional uses and research on pharmacological properties of this plant has mostly focused, however there is limited evidence about its effectiveness as a feed additive for broiler chickens. Researchers can get valuable insights by resolving this inadequacy and would be able to promote more sustainable and health-oriented techniques in poultry farming. Subsequently, this will assist in managing the emergence of antibiotic-resistant bacteria and contribute in achieving the primary goal of reducing dependence on antibiotics (Husain et al., 2021). Manufacturing of antibiotic-free broiler will be vibrant step in both animal and public health. Sustainable and efficient chicken production might be achieved by exploring natural feed additives like *J. sambac*. Therefore, the current study aimed to determine the impact of *J.sambac* leave powder extracts on broilers growth performance, immunological function and coliform bacterial levels.

## Materials and methods

### Ethical consideration

All animal experimental procedures were approved by the Animal Ethics Committee of Shaheed Benazir Bhutto Women University Peshawar (protocol code SBBWUP20230037, August 2023).

### Processing of *Jasminum sambac* leaves

*Jasminum sambac* leaves were collected from Charsadda region of Pakistan in June. After harvesting fresh leaves, they were properly cleaned with tap water to remove dirt and dried for 11 days in the shade. Dried leaves were grinded into a fine powder, and about 50 grams of leave powder were infused in hot boiling water overnight. Following that, the infusion was filtered via filter paper, and the filtrate was given to the chicks in accordance with the prescribed treatment doses.

### Animal housing and experimental setup

Total 100 (Ross) broiler chickens were randomly divided into four groups, with each group including 25 birds. The birds were raised in cages and fed a balanced commercial diet until the end of the trial (Table 1), and were kept in 20 cages, with five birds per cage. Each cage was 100 cm long, 80 cm wide, and 50 cm high, giving each bird 1,600 cm² of floor space. On the first day, lighting was provided for 24 hours, and thereafter every day for 23 hours. The birds' overall environment, including temperature and humidity, met the requirements of normal brooding management for the raising stages, as outlined in the Ross Broiler Management Manual (Ross, 2002). The 1st group was designated as control group (C) and was provided with a regular meal devoid of any additional supplements. A diet enriched with 3% Jasminum sambac leaf powder (JSLP 1) was given to 2nd group. The 3rd group was administered with diet containing 6% Jasminum sambac leaf powder (JSLP 2), whereas the 4th group was provided with a diet containing 9% Jasminum sambac leaf powder ((JSLP 3).

**Table 1:** Composition of the basal diet provided to broilers during the experiment.

**Ingredients**	**Amount**
Corn %	43.3
Wheat barn %	38.6
Crude protein %	22.5
Digestible energy kcal/kg of diet	2500
Calcium %	1.1
Phosphorus %	0.45
Lysine %	0.93
Methionine and Cysteine %	0.78

### Growth performance

Body weight (BW), weight gain (WG) and feed intake (FI) was monitored daily and the feed conversion ratio (FCR) was determined using the following formula as described by (Quaye et al., 2023).

$$\text{FCR}=\frac{\text{Total}\;\text{feed}\;\text{consumed}\;\text{in}\;\text{Kg}}{\text{Total}\;\text{weight}\;\text{gain}\;\text{in}\;\text{Kg}}$$

#### Analysis of Coliform bacterial count

The total coliform bacterial count from fecal samples was analyzed on days 21 and 42 of the experiment after slaughtering 2 animals (healthy) from each group as described by (Raheem et al., 2012). Aseptic containers were used to collect specimens and kept at low temperatures 4°C, and transported to the laboratory for examination within 24 hours. Repeated 10-fold dilutions such that 10^7^ were performed in 9ml of 0.1% of sterile peptone solution to count the total coliform bacteria (CFU/ml) and culture was obtained on MacConkey agar plates. Incubation of plates was performed at a temperature of 37°C for a duration of 24 hours. The number of red colonies on the agar plates, indicative of coliform bacteria, was recorded."

$$\text{Colliform}\;\text{count}\;\left(\text{CFU}/\text{ml}\right)=\frac{\text{Number}\;\text{of}\;\text{Colonies}\;\times \;\text{dilution}\;\text{factor}}{\text{Volume}\;\text{of}\;\text{culture}\;\text{plated}\;\text{in}\;\text{ml}}$$

#### Hematological mMeasurements

Blood samples were obtained at days 14, 28, and 42 from the brachial vein of healthy chickens. A total 2ml of blood from 2 birds from each group was drawn into tubes containing ethylene diamine tetra-acetic acid (EDTA). The samples were immediately stored at -20°C for further analysis. The Sahli “acid hematin method” was employed to determine the concentration of haemoglobin by using hemocytometer (Ananya et al., 2014). Haemoglobin was converted into acid haematin by adding a drop of blood into a graduated tube containing hydrochloric acid (HCL) and was diluted with drops of water until it matches the color of the tinted comparator glass. The haemoglobin level was estimated by reading the value (one decimal point in grams per deciliter) directly from the scale on Sahli's tube according to the height that the final solution has reached. The RBC counts were determined using a Neubauer chamber in the hemocytometer.". For counting red blood cells (RBCs), the blood was combined with Hayem's solution to generate a diluted specimen and observed under 40x objective Olympus CX21 phase microscope.

The total cell count was determined by applying the following formula:

$$\text{Total}\;\text{cells}\;\left(\text{per}\;mm^{2}\right)=N\;\times \;50\;\times \;200$$

where N is the entire number of cells in the 80 small squares.

#### Analysis of immunological response

To assess the immunological response, differential leukocyte count (DLC) was performed. For this purpose, the chickens were randomly selected, and blood was collected from 2 chickens per treatment group to assess DLC. A thin smear of each blood sample was created immediately on glass slides and were stained subsequently with Leishman's stain. Each slide's smear was allowed to air dry, and a drop of before an immersion oil drop was applied and viewed under a microscope (40x magnification) (Ananya et al., 2014). The white blood cells and absolute counts for various types of leukocytes were computed individually by dividing relative count of that type × total WBC count/ 100).

### Computational analysis of data

All data were analyzed using SPSS-IMB (version 20). One-way analysis of variance (ANOVA) was used to evaluate the impact of the different treatments over a period of time. Statistical significance was determined; the means were compared using the Duncan's New Multiple Range Test (Duncan's, 1955) at a p < 0.05. The results were shown as the mean value ± standard error of the mean (SEM).

## Results

### Broiler performance

The effect of Jasminum sambac leaf powder (JSLP) on broiler performance is summarized in Table 2. During the initial growth phase (1–14 days), no significant differences (P>0.05) were observed in body weight, weight gain, feed intake, or feed conversion ratio (FCR) between the control and JSLP groups. However, during the subsequent growth periods (14–28 days and 29–42 days), the addition of JSLP to the broilers' diet resulted in a significant increase (P<0.05) in body weight and weight gain. Over the entire 42-day experiment, the JSLP group exhibited significantly higher (P<0.01) body weight, weight gain, and feed intake compared to the control group, although no significant differences in FCR were observed (P>0.05).

**Table 2:** Effect of Jasminum sambac on broiler performance.

Variable	Groups			
	Control (0)	JSLP (1) 3%	JSLP (2) 6%	JSLP (3) 9%
Day 0				
Body weight (g)	50±0.37	45±1.048	42.8±0.73	48.4±0.812
Weight gain	33.9±0.35^c^	42.9±1.06	45.8±0.63^a^	47.4±0.831^a^
Feed intake	33±0.37	32.8±0.748^c^	28.2±0.734^b^	34±0.707a
FCR	0.66±0.004	0.732±0.033	0.654±0.016	0.7±0.023
Day 14				
Body weight (g)	302±0.31	308.6±0.510	304.2±0.374	306.8±1.019^a^
Weight gain	252±0.4	264.2±0.969^b^	261.2±0.538	258.4±0.927^a^
Feed intake	290±0.4	301.6±0.509^b^	291.8±2.437^a^	291.6±0.812^a^
FCR	0.96±0.003^c^	0.974±0.002	0.956±0.009^c^	0.95±0.003
Day 28				
Body weight (g)	1100±1.85	1191±2.073	1371.4±0.871^a^	1749±1.140
Weight gain	798±0.37	881±2.501	1067.2±0.860^a^	14422±1.319^a^
Feed intake	980±0.44	1048.6±3.203^b^	1253.2±1.240^a^	1589.2±0.860^a^
FCR	0.89±0.003^c^	0.876±0.002	1.172±0.002	0.906±0.002
Day 42				
Body weight (g)	2240±0.58	2258±2.073	2450±2.449^a^	2668.6±0.748^a^
Weight gain	1140±0.24	1067±2.828	1079±2.549	922±2.509
Feed intake	2180±1.15^b^	2179.6±1.326	2386.6±3.187	2589.2±2.853^a^
FCR	0.97±0.0031	0.964±0.002^c^	0.976±0.002	0.964±0.002

The means with different superscript in same rows are statistically different from each other (P < 0.05). ± Standard error mean

### Gut bacterial count

The findings on the counts of coliform bacteria in experimental chickens on days 21 and 42 are shown in Table 3. The appropriate range for coliform bacteria is between 3.0 and 30.0. The number of coliform bacteria was notably decreased (P<0.01) on day 21. There was a significant decline in the number of coliform bacteria by day 42.

**Table 3:** Coliform bacterial count (× 10⁶) in broiler chickens consuming Jasminum sambac.

Variable	Groups			
	Control (0)	JSLP (1) 3%	JSLP (2) 6%	JSLP (3) 9%
		Day 21		
CBC (× 10⁶ CFU/ml)	9.88±0.224^c^	7.72±0.472^b^	7.01±0.430^a^	7.12±0.316^a^
		Day 42		
CBC (× 10⁶ CFU/ml)	10.29±0.143^c^	8.40±0.543^b^	6.98±1.237^a^	7.10±0.519^a^

The means with different superscript in same rows are statistically different from each other (P < 0.05). ± Standard error mean

### Hematological measurements

**Fig. 1:**
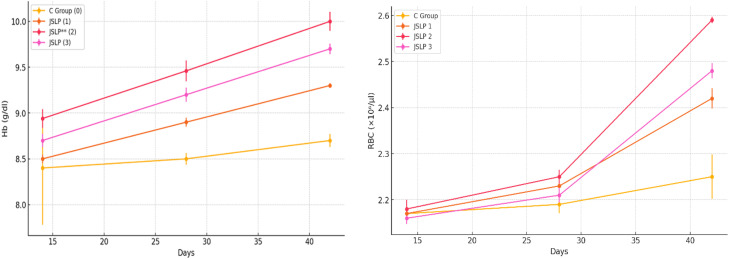
The hemoglobin levels and average RBC values of broiler chickens consuming Jasminum sambac on days 28, and 42, for different treatment groups. The error bars indicate the standard error for each data point.

After JSLP supplementation, hematological alterations were shown in Fig. 1. These variations included shifts in haemoglobin (Hb) concentration and red blood cell (RBC) count. Between groups, there were a significant difference (p<0.05) in the average Hb concentration and RBC count on days 14 and 42. In comparison to the control and other treatment groups, the JSLP 2 group, which received 6% of JSLP, showed noticeably greater Hb and RBC counts. Nevertheless, there was no substantial disparity observed in haemoglobin concentration and red blood cell count between the JSLP 1 and JSLP 3 groups.

### Immunity parameters

We observed the effects of Jasminum sambac leaf powder (JSLP) on the counts of different types of white blood cells in broiler chickens on day 42 (Table 4). The study observed statistically significant elevations (P<0.05) in neutrophils, lymphocytes, monocytes, eosinophils, and basophils in the JSLP 2 group as compared to both the control group and the other treatment groups. Leukocyte counts have changed, and these shifts suggest that broilers given JSLP supplementation have a stronger immunological response. The notable rise in neutrophils indicates an enhanced ability to combat bacterial infections and inflammation, as neutrophils are crucial phagocytic cells that play a vital role in the initial defense against microbial invaders. The elevated level of lymphocyte counts indicates a strong adaptive immune response, improving the broilers' capacity to generate antibodies and combat infections with greater efficiency.

**Table 4:** Impact of consuming *Jasminum sambac* on Differential leukocyte counts in broiler chickens on day 42.

Parameters		Treatments		
	Control (0)	JSLP (1) 3%	JSLP (2) 6%	JSLP (3) 9%
Neutrophil (%)	48±1.048	54±1.077^a^	59±0.489	55±0.894^b^
Lymphocyte (%)	25±1.304	35±0.948	38±0.748^a^	35±0.707^a^
Monocyte (%)	3±0.316^c^	6±0.547^b^	7±0.447^a^	6±0.316
Eosinophil (%)	2±0.245^c^	3±0.316	4±0.244^b^	3±0.374^a^
Basophil (%)	1±0.058	1±0.054	1±0.04^b^	1±0.058

The means with different superscript in same rows are statistically different from each other (P < 0.05). ± Standard error mean

The results support the idea that addition of the JSLP supplementation in the diet of broiler chickens appears to strengthen their immune systems, which may improve their resilience to a range of illnesses and infections. After experimentation, JSLP is considered as a valuable supplement in poultry nutrition and health management because of its ability to improve the health, immune system, and productivity of broiler chickens. As we know higher eosinophil numbers suggest an enhanced immune reaction to parasite infections and allergic reactions, on the other hand and increase in basophils indicates an improved capacity to regulate allergic responses and inflammation. In this research, results found the elevated monocyte counts in the JSLP 2 group indicates an enhanced ability for phagocytosis and removal of debris, which is essential for the control of chronic infections and inflammation.

## Discussion

The supplementation JSLP in broilers diet resulted in a significant (P<0.05) increase in body weight and weight gain of the chickens. The overall results of the growth experiment (1-42 days) clearly demonstrated that the addition of JSLP as growth promoter into chickens’ diet caused a significant improvement in the productive parameters of the broilers. The body weight, weight gain and feed intake of broilers in JSLP group were significantly (P<0.01) higher as compared to the control group which is in line with previous findings (Quaye et al., 2023).

There might be many factors that cause variation in the outcomes, might be explained by differences in the food components, experimental setups, and the specific bioactive compounds in JSLP that enhance nutrient absorption and breakdown. On the other hand, our feed conversion ratio (FCR) study's results indicate that adding JSLP, like Saccharomyces cerevisiae, positively impacts weight gain without significantly altering FCR (Dumorne et al., 2020). In this study we also revealed that the mortality rates of broilers in the JSLP group were significantly lower than those in the negative control group (P<0.05). This possibility might be because of immunostimulant effects of JSLP that may be the cause of decreased mortality, as evidenced by the significantly higher levels of neutrophils, monocytes, lymphocytes, eosinophils, and basophils in the JSLP groups (P<0.002). which is consistent with the reported higher immune responses in broilers given herbal therapy (Salahiddin et al., 2006; Rafique et al., 2020).

This research reported improvements in blood parameters with the use of several dietary supplements which is similar to the previous findings (Durrani et al., 2008; Elnaggar et al., 2022). It is also observed that the Hb and RBC counts in the treatment groups were greater than those in the control group (P<0.01). JSLP 2 group (6%) showed a substantial improvement in haematological parameters, such as haemoglobin (Hb) concentration and red blood cell (RBC) count.

It has been revealed that phytogenic bioactive compounds have the ability to decrease the number of harmful bacteria in the intestine by inhibiting their attachment to the mucosal lining (Hamady et al., 2015; Sugiharto et al., 2020). In this research we also found that the impact of JSLP on coliform bacterial levels in the intestines of broilers was significant. Pathogenic Coliform bacterial counts considerably decreased (P<0.01) on day 21 and 42 in the JSLP groups. The reason behind it is that Jasminum sambac's essential oil and methanol extract exhibit antibacterial and antioxidant properties (Shehkar and Prasad, 2015). Our results are consistent with the earlier study (Khalaji et al., 2021), which found that adding Artemisia leaves to broiler diet lowers the coliform population.

It was also evident that the supplementation of JSLP resulted in increased levels of haemoglobin and red blood cell counts, especially in the group that received 6% JSLP. This indicates an improvement in general health and the ability to carry oxygen. Antibacterial characteristics of JSLP is clearly evident from the presence of fecal coliform bacteria in the treated groups. Considering the favorable effects on growth performance and health indicators, it is advisable to use JSLP in broiler diets. We hope that future research into the effects of JSLP on nutrient absorption, meat quality and potential long-term consequences in the human food chain can be elucidated. This study demonstrates that extended consequences of JSLP supplementation on the efficiency and well-being of broiler chickens should be examined. If you apply those above suggestions quality of broiler chickens would be in a good position and best practices are followed to achieve optimum growth performance with high returns. This in turn could tackle antibiotic resistance and promote sustainable farming practices.

### Limitations

Jasminum sambac leaf powder was only evaluated on Ross broilers, which limits the findings' applicability to other chicken breeds that could react differently to the treatment. Furthermore, the underlying mechanisms by which JSLP influences immunological response, growth performance, and coliform reduction are not entirely understood, which restricts our comprehension of how these advantages are accomplished.

## Conclusion

This study uses J. sambac leaf powder (JSLP) to assess its effect on growth and immune parameters of broiler chickens. It has been shown that that supplementing JSLP to the food of broiler chickens has a considerable positive effect on their development, blood characteristics, and immune system. Additionally, the above-mentioned feed additives enhance blood counts (p<0.05) and strengthen broilers' immune systems (p<0.001). This research might give an edge to the broiler industry to use plant feed as an alternative to antibiotics, which will be cost effectives in raising healthy broilers on a larger scale. However, further studies are needed to shed light on the mechanism through which these additives enhance broilers health.

## Disclosures

The authors declare no conflicts of interest. The manuscript has been "spell-checked" and "grammar checked." All references mentioned in the Reference List are cited in the text.

## Declaration of competing interest

The author(s) declare no conflict of interest
